# Editorial: Case reports in thoracic oncology: 2022

**DOI:** 10.3389/fonc.2024.1369799

**Published:** 2024-03-20

**Authors:** Yaron Perry

**Affiliations:** ^1^ Division of Thoracic Surgery, Buffalo General Hospital, Buffalo, NY, United States; ^2^ Division of Thoracic Surgery, Erie County Medical Center, Buffalo, NY, United States; ^3^ Department of Surgery, Jacobs School of Medicine and Biomedical Sciences, University at Buffalo, Buffalo, NY, United States

**Keywords:** case report, thoracic oncology, unique findings, novel therapies, unique pathology

When I was asked by the editorial board of Frontiers to help edit a whole Research Topic on “*case reports in thoracic oncology*,” I was caught by surprise. In an era of large databases and unlimited access to medical information, is there still a role for case reports?

Physicians began describing interesting cases involving all specialties in the early days of humanity, Ancient Egyptian medicine (c. 1600 BCE) has papyrus records of case reports as well as reports by Hippocrates (460–370 BCE) (1). Case reports describe important scientific observations that are missed or undetected in clinical trials and provide individual clinical insights. A case report of Kaposi’s sarcoma in a young homosexual man was the initial main observation to the finding of acquired immune deficiency syndrome (2). In 1817, James Parkinson wrote an article titled “An essay on the shaking palsy,” later it lead to the discovery of the disease carrying his name (Parkinson’s disease) (3). Case reports linked the anorexic agents fenfluramine and dexfenfluramine with primary pulmonary hypertension led to clinical trials that investigated the mechanism and incidence of this adverse effect, causing their withdrawal from the market (4, 5). Hemolytic-uremic syndrome is a severe condition associated with Escherichia coli and characterized by hemolytic anemia, thrombocytopenia, and acute renal failure. At the start of an outbreak, a case series reported dramatic resolution of symptoms of Escherichia coli-associated hemolytic-uremic syndrome after treatment with monoclonal antibody eculizumab (6). This case series led to the adoption of eculizumab as a treatment option. In 1985, the American Medical Association reprinted 51 papers from the Journal of the American Medical Association that had significantly changed the science and practice of medicine during the 150 years of the organization’s existence (7); five of these papers were case reports.. But in the last decade, most professional journals and scientific editorial committees are publishing mainly case studies, double-blind control studies, cohort studies, large database reports, and original scientific work, while case reports are scattered and usually limited to a single page of imaging or rare occasions and account for less than 2% of the journal content. In the second half of the 20^th^ century, the significance of a case report as a type of research article was downgraded due to low ranking in the evidence hierarchy (8).

As a thoracic surgeon, I recently admitted a young patient to our thoracic surgery service: a very fit 27-year-old police officer who initially presented with pneumonia and parapneumonic effusion. Within a matter of hours, his condition deteriorated so that he needed mechanical ventilation and pressors support. He was treated by multiple medical teams from internal medicine, infectious disease, and intensive care and multiple surgeons; he was stabilized and treated with a chest drain. Because he was not improving, I looked through the literature and found a case report describing the same clinical picture with isolation of very rare bacteria—*Streptococcus constellatus* (9). The patient in the described case report was treated aggressively with surgical exploration and drainage of the pleural space with decortication, leading to expansion of the lung and recovery. The initial Gram stain of cultures from our patient were indicative of *Streptococcus* but without the final species isolation. On the basis of the case report, I took the patient to the operating room and performed an extensive decortication of his pleural space with expansion of the lung and clearance of infected tissue and fluid, which ultimately resulted in complete recovery of the patient within 48 hours. The final cultures documented that the bacterium was the rare *Streptococcus constellatus*.

Having learned from a case report, which led to saving my patient’s life, I was very enthusiastic to contribute and collect many case reports from my colleagues in the thoracic oncology field. Publication of their experiences with rare cases will hopefully contribute to the care of patients with rare manifestations in the future.

We carefully collected case reports, such as a case that describes a rare/unconventional treatment from a group in the Department of Radiology, The First Medical Center, Chinese People’s Liberation Army (PLA) General Hospital, Beijing, China; they describe radiofrequency ablation combined with biopsy for Cushing’s syndrome due to ectopic adrenocorticotropic hormone lesions in the lung (this Research Topic). We included an article describing a new conventional treatment with rare side effects from a group in the Department of Pharmacy, Zhongnan Hospital of Wuhan University, Wuhan, China; they describe liver injury associated with dacomitinib (this Research Topic). Clinicians should pay particular attention to the possibility of drug-induced liver injury during dacomitinib treatment. A group from the Department of Pathology, Peking University Shenzhen Hospital, Shenzhen, China, described a rare manifestation of a known disease (this Research Topic): a case of a stroma-rich variant of Castleman’s disease of the hyaline-vascular type featuring atypical hyperplasia of the stromal cells and malignant behavior. In addition, a group from the Department of Thoracic Oncology, State Key Laboratory of Biotherapy, Cancer Center, West China Hospital, Sichuan University, Chengdu, China, report on the efficacy of new targeted immunotherapy in lung cancer (this Research Topic). In this research, a patient with non-small cell lung cancer with primary SDN1-BRAF fusion responded well to continuous trametinib monotherapy. Further research is required to understand the biochemical and oncogenic mechanisms and identify the targeted strategy in NSCLC patients harboring BRAF fusions, but this case may lead to a new mode of treatment.

Treatments such as insulin for diabetes and cholesterol-lowering drugs for coronary heart disease first appeared as scattered case report descriptions in the literature. Their subsequent persistence in the literature and scientific trials with expansion to larger patient populations led these novel therapies to become the standard of care. In the same way, the field of thoracic oncology can benefit from novel targeted therapies that are rapidly evolving. The publishing of case reports in thoracic oncology will increase descriptions of new diseases from which clues to etiology can be derived. Indeed, the first clues about tobacco smoking and lung cancer came from surgical patient series in the 1920s and 1930s, though formal case-control and cohort studies came only decades later (10). Case reports can lead to new therapies, recognition of side effects, or as in my patient, serve to educate. Case histories are like a lesson from the clinicopathological conference: “do not make the same mistake I did.” Cases with an unfavorable outcome can be collected to see whether that outcome might have been prevented (11).

By giving this platform to many clinicians in the field of thoracic oncology to publish their unique findings, I hope that many novel therapies will be established for the care of our future patients.

This is what it is all about.

I hope you will find these case reports informative and helpful.

Happy New Year!

## Author contributions

YP: Conceptualization, Writing – original draft, Writing – review & editing.
